# Bradykinin 1 receptor blockade subdues systemic autoimmunity, renal inflammation, and blood pressure in murine lupus nephritis

**DOI:** 10.1186/s13075-018-1774-x

**Published:** 2019-01-08

**Authors:** Ling Qin, Yong Du, Huihua Ding, Anam Haque, John Hicks, Claudia Pedroza, Chandra Mohan

**Affiliations:** 1 Department of Nephrology & Rheumatology, Shanghai Tenth People’s Hospital, Tongji University School of Medicine, Shanghai, People’s Republic of China; 2 Department of Biomedical Engineering, University of Houston, 3605 Cullen Boulevard, Houston, TX 77204 USA; 3 Texas Children’s Hospital, Houston, TX USA; 4 University of Texas at Houston, Houston, TX USA

**Keywords:** Bradykinins, Hypertension, Lupus nephritis, Kallikrein

## Abstract

**Objective:**

The goal of this study was to explore the role of bradykinins and bradykinin 1 receptor (B1R) in murine lupus nephritis.

**Methods:**

C57BL/6 and MRL/lpr mice were compared for renal expression of B1R and B2R by western blot and immunohistochemistry. MRL/lpr lupus-prone mice were administered the B1R antagonist, SSR240612 for 12 weeks, and monitored for blood pressure, proteinuria, renal function, and serum autoantibodies.

**Results:**

Renal B1R:B2R ratios were significantly upregulated in MRL/lpr mice compared with B6 controls. B1R blockade ameliorated renal pathology lesions, proteinuria, and blood pressure, accompanied by lower serum IgG and anti-dsDNA autoantibody levels, reduced splenic marginal zone B cells and CD4^+^ T cells, and renal infiltrating CD4^+^ T cells, macrophages, and neutrophils. Both urine and renal CCL2 and CCL5 chemokines were also decreased in the B1R blocked MRL/lpr mice.

**Conclusion:**

Bradykinin receptor B1R blockade ameliorates both systemic immunity and renal inflammation possibly by inhibiting multiple chemokines and renal immune cell infiltration. B1R blockade may be particularly attractive in subjects with concomitant lupus nephritis and hypertension.

**Electronic supplementary material:**

The online version of this article (10.1186/s13075-018-1774-x) contains supplementary material, which is available to authorized users.

## Background

Systemic lupus erythematosus (SLE) is a chronic systemic autoimmune disorder characterized by the production of autoantibodies, multiple organ involvement, and diverse clinical manifestations. Lupus nephritis (LN) is one of the commonest and most severe clinical features of SLE and leads to significant morbidity and mortality. Although 5- and 10-year SLE/LN survival rates have improved in recent years, significant challenges exist in understanding the pathogenesis of LN and designing appropriate therapy.

Kinins are generated from kininogens catalyzed by kallikreins. Kinins exert their biological functions through two types of bradykinin receptors: B1R and B2R. B1R is expressed at inflammatory sites, whereas the B2R is expressed in healthy tissue constitutively. It has been reported that B1R is involved in inflammation, pain, and fibrosis induced by inflammatory mediators. Indeed, this receptor-mediated pathway has been implicated in inflammatory bowel disease, vasculitis, experimentally induced nephritis, and acute gout [1–4]. In resonance with these reports, it has been shown that B1R antagonism or ablation plays a protective role in nephrotoxic serum-induced glomerulonephritis [5], lipopolysaccharide (LPS)-mediated acute renal inflammation [6], and experimental obstructive nephropathy [7]. However, the renal expression of B1R and its exact role in the pathogenesis of LN are poorly investigated. This is particularly important given that bradykinins are elevated in SLE [8]. This study was designed to explore the effect of B1R blockade on murine LN and to understand the underlying mechanisms.

## Materials and methods

### Mice and B1R blockade

Female C57BL/6 (B6) and MRL/lpr mice were purchased from the Jackson Laboratory (Bar Harbor, ME, USA) and maintained in a specific pathogen-free colony. Animal experiments were approved and conducted in accordance with University of Houston’s Institutional Animal Care regulations. Twenty-eight 4-month-old MRL/lpr mice were divided into a control group (*n* = 14) and a treatment group (n = 14) randomly for the *in vivo* studies. The B1R antagonist SSR240612 was purchased from Adooq Bioscience (Irvine, CA, USA). SSR240612 was dissolved in water containing dimethyl sulfoxide (DMSO) to make a final concentration of 1.5 mg/mL in 0.9% DMSO. Mice in the treatment group were administered 10 mg/kg per day SSR240612 by gavage every other day, whereas the mice in the control group received 10 mg/kg per day 0.9% DMSO by gavage every other day; 24-h urine was collected using metabolic cages from all mice. Blood and urine were collected at 0, 8, and 12 weeks after treatment to assess proteinuria, serum blood urea nitrogen (BUN), alanine aminotransferase (ALT), and aspartate aminotransferase (AST). At 12 weeks after treatment, all mice were euthanized by using a CO_2_ chamber and cervical dislocation.

### Blood pressure measurement

Blood pressure (BP) was monitored before and after 12 weeks of treatment using a non-invasive mouse-rat BP monitor (CODA, Kent Scientific, Torrington, CT, USA). The mean artery pressure (MAP) was used to compare the BP in the two groups.

### Renal histopathology

Renal tissue was prepared as 4-μm sections followed by formalin-fixation, dehydration, and paraffin-embedding. Slides were stained with hematoxylin and eosin or periodic acid–Schiff (PAS). Pathological changes in glomeruli, tubules, or interstitial areas were examined in a blinded fashion by a pathologist. Glomerulonephritis severity was graded on a 0–4 scale in 20 glomeruli as follows: 0, normal; 1, mild increase in mesangial cellularity and matrix; 2, moderate increase in mesangial cellularity and matrix, with thickening of the glomerular basement membrane (GBM); 3, focal endocapillary hypercellularity with obliteration of capillary lumina and a substantial increase in the thickness and irregularity of the GBM; 4, diffuse endocapillary hypercellularity, segmental necrosis, crescents, and hyalinized end-stage glomeruli. The interstitial score was determined by examining 20 high-power fields, and interstitial inflammation was scored on a scale from 0 to 4 as follows: 0, no lesions; 1, mild focal dilation or few foci of tubular atrophy or both; 2, larger numbers of dilated tubules with widening of interstitium or larger numbers of foci of tubular atrophy or both; 3, extensive dilation of tubules with cyst formation and widening of interstitium or a large numbers of foci of tubular atrophy or both; 4, extensive tubular atrophy [9]. Glomeruli with any degree of sclerosis or collapse and thrombonecrotic lesions were graded on a 0–4 scale, corresponding to absence of lesions, or involving less than 10%, 11–20%, 21–30%, or more than 31% of glomeruli, respectively.

### Immunohistochemistry

Five B6 mice and five MRL/lpr mice at 4 months of age were used to assess renal expression of B1R and B2R. Kidney sections obtained from 4-month-old C57BL/6 J and MRL/lpr mice were stained with the following primary antibodies: rabbit anti-mouse B1R antibody (Bioss Inc., Woburn, MA, USA) and rabbit anti-mouse B2R antibody (Bioss Inc.). Antigen retrieval was performed by using a sodium citrate buffer (10 mM sodium citrate, pH 6.0) in a microwave oven, protein-blocked for 20 min, and endogenous enzyme-blocked for 20 min, followed by incubation with dextran polymer conjugated with horseradish peroxidase (HRP) and affinity-isolated immunoglobulins, using diaminobenzidine (DAB) + as chromagen. All reagents were purchased from Dako (Santa Clara, CA, USA).

### Western blot

Western blot was performed as described previously [10]. In brief, total renal protein was extracted and prepared in sample buffer by boiling for 10 min. Samples were spun down, subjected to SDS-PAGE, and transferred to a PVDF membrane using a Bio-Rad Trans-Blot Turbo transfer system. Rabbit anti-mouse B1R antibody (Bioss Inc.), rabbit anti-mouse B2R antibody (Bioss Inc.), and anti-α-tubulin (Cell Signaling Technology, Beverly, MA, USA) were used as the primary antibodies. HRP-conjugated secondary antibodies and the ECL-plus detection kit (Amersham, Little Chalfont, UK) were used for western blot. For analysis, bands were quantified by ImageJ^®^.

### Flow cytometry

Animals were sacrificed and the spleens and kidneys were collected for flow cytometry analysis. The monoclonal antibodies used for splenic flow cytometry were CD4-PE, CD3-PE-cy7, Foxp3-FITC, CD69-percp-cy7, B220-PE-cy7, CD21-FITC, CD23-PE, CD11c-PE-cy7, CD11b-APC, F4/80-PE, CD86-FITC, and F4/80-PerCP. The monoclonal antibodies used for renal flow cytometry were CD4-PE, CD3-Percp, Foxp3-FITC, CD45-APC-cy7, CD11b-FITC, CD11c-PE-cy7, F4/80-PE, and Gr-1-Percp (eBioscience, Hanover Park, IL, USA). Cell counting was performed by using a Cellometer^®^ automated cell counting system (Sigma-Aldrich, St Louis, MO, USA) for absolute cell numbers. The Novocyte flow cytometer system (ACEA Bioscience Inc., San Diego, CA, USA) was used for flow cytometry, and analysis was performed as described [11]. Data were analyzed using Novocyte software (ACEA Bioscience Inc.). At least 200,000 events were acquired for each analysis.

### Total serum IgG and autoantibody detection

Total serum IgG was determined by using commercial enzyme-linked immunosorbent assay (ELISA) kits in accordance with the instructions of the manufacturer (eBioscience, San Diego, CA, USA). In brief, anti-mouse IgG was first coated onto plates and blocked. Test samples were diluted serially and added to the plates for 2 h at room temperature, followed by incubation with detection antibody, substrate solution, and stop solution. Concentrations were determined by using a standard curve.

ELISA assay for anti-dsDNA, ssDNA, and histone autoantibodies was performed as described previously [12]. Briefly, Immulon 2B plates were pre-treated with 1% mBSA in phosphate-buffered saline, and 50 μg/mL dsDNA or ssDNA (or histone) was added, and blocked, before adding a 1:400 dilution of mouse serum. All autoantibodies were detected with HRP-conjugated goat anti-mouse IgG, and the plates were read at 405 nm. All reagents were obtained from Sigma-Aldrich. Pooled serum from lupus-afflicted MRL/lpr mice with a starting dilution of 1/100 served as the standard. These absorbance values were fit to derive a curve, using a four-parameter fit, and all samples’ absorbance values were converted to arbitrary units by using this standard curve.

### ELISA for urine or kidney lysate chemokines

In this study, urine and kidney lysate CCL2, CCL5, and CXCL9 levels were measured by using ELISA kits from R&D Systems (Minneapolis, MN, USA) in accordance with the instructions of the manufacturer. Briefly, diluted urine or kidney lysate samples were added to capture antibody pre-coated 96-well microplates. After incubation with samples, the detection antibody was added, followed by streptavidin-HRP, and substrate. A microplate reader ELX808 from BioTek Instruments (Winooski, VT, USA) was used to read the optical density at 450 nm. The concentration was calculated based on a standard curve. Urinary creatinine concentrations were determined by using Creatinine Parameter Assay Kit (R&D Systems). Urine creatinine concentrations were used to normalize urine chemokine concentrations.

### Statistics

Data were analyzed and plotted by using GraphPad Prism 5 software (GraphPad Software, San Diego, CA, USA). The Kolmogorov–Smirnov test was used to assess the normality of the data. For comparison between two groups, the *t* test was used when the normality test passed; otherwise, a non-parametric Mann–Whitney test was used to analyze the data. A two-tailed *P* value of less than 0.05 was considered significant. For all data, the statistical results were also re-computed after correcting for the missing data due to the deceased animals (by assigning to the deceased mice the mean values recorded for the respective phenotypes in the surviving mice in the treatment group or control group). These corrected *P* values are appended to the legends of Figs. 3, 4, and 5.

## Results

### Renal expression of B1R and B2R in MRL/lpr mice assayed using two complementary approaches

**Fig. 1:**
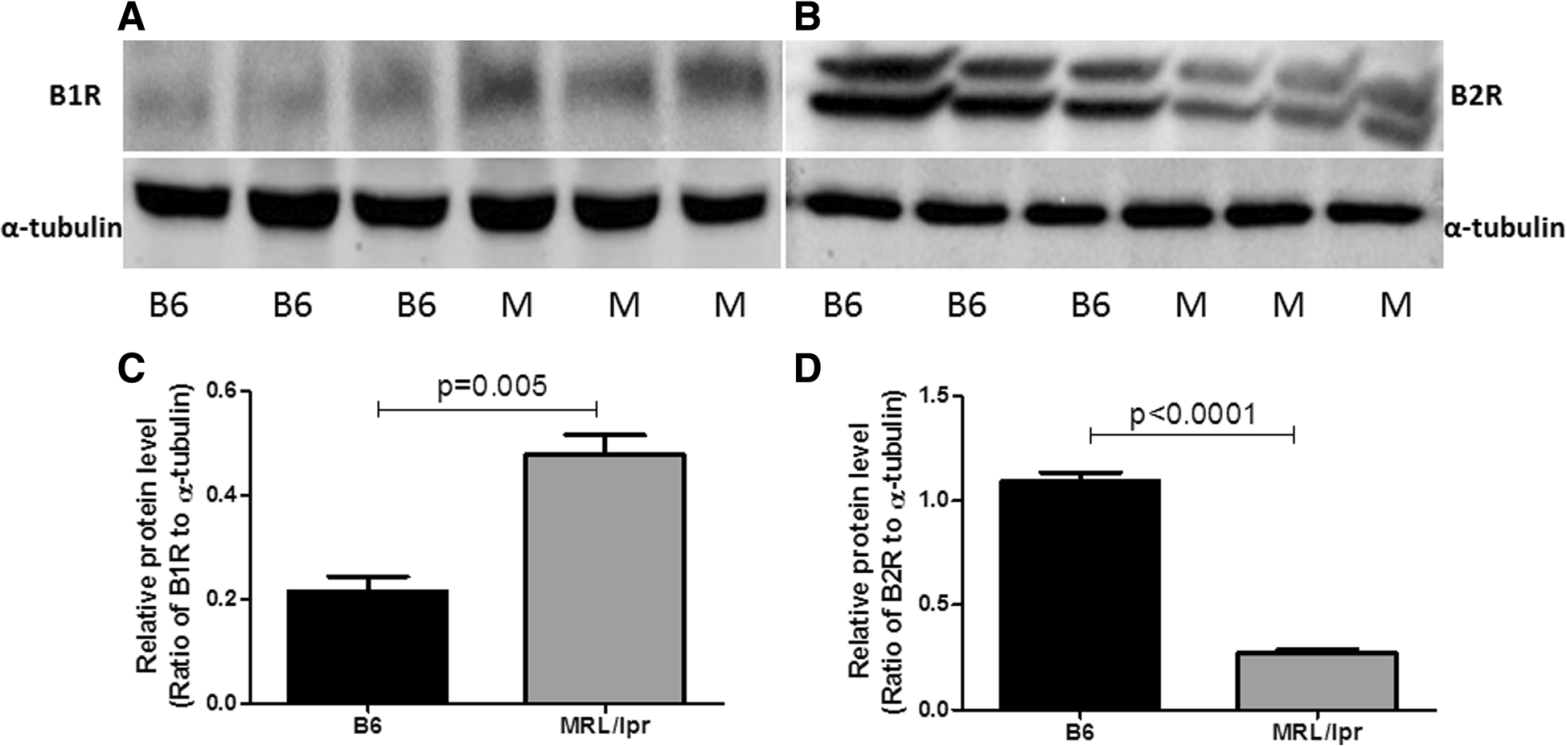
Renal bradykinin 1 receptor (B1R) and B2R expression in MRL/lpr mice and B6 mice as assessed by western blot analyses. Rabbit anti-mouse B1R antibody (Bioss Inc., Woburn, MA, USA), rabbit anti-mouse B2R antibody (Bioss Inc.), and anti-α-tubulin (Cell Signaling Technology, Beverly, MA, USA) were used as the primary antibodies. Horseradish peroxidase (HRP)-conjugated secondary antibodies and the ECL-plus detection kit (Amersham, Little Chalfont, UK) were used for western blot. Western blot analysis revealed that renal B1R expression was increased in MRL/lpr mice compared with B6 mice (**a**, **c**) but that B2R expression was decreased in MRL/lpr mice compared with B6 mice (**b**, **d**). Shown data are representative of blots from five B6 mice and five MRL/lpr mice

**Fig. 2:**
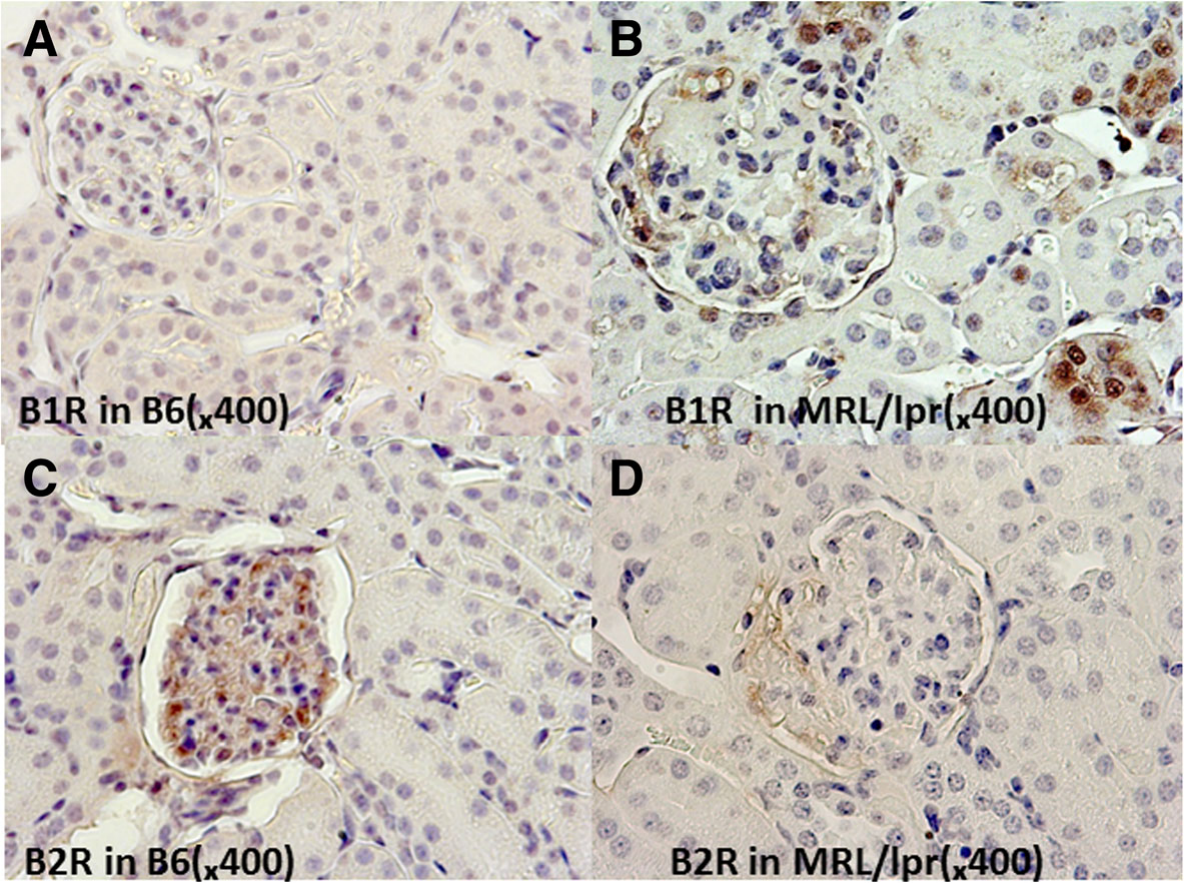
Renal bradykinin 1 receptor (B1R) and B2R expression in MRL/lpr mice and B6 mice as assessed by immunohistochemistry (IHC). IHC was used to monitor renal B1R and B2R expression in control and lupus mice. Kidney sections obtained from 4-month-old C57BL/6 J and MRL/lpr mice were stained with the following primary antibodies: rabbit anti-mouse B1R antibody (Bioss Inc. Woburn, MA, USA) and rabbit anti-mouse B2R antibody (Bioss Inc. Woburn, MA, USA). IHC analysis indicated elevated renal B1R expression in MRL/lpr mice (**b**) compared with B6 mice (**a**). B6 mice demonstrated stronger renal B2R expression than MRL/lpr mice (**c**, **d**). Shown data are representative of IHC staining from five B6 mice and five MRL/lpr mice

To explore the renal expression of bradykinin receptors B1R and B2R in MRL/lpr mice, total renal protein was extracted and examined by western blot. Renal B1R expression was increased in MRL/lpr mice compared with C57BL/6 J mice (Fig. 1a, c), whereas renal B2R expression was decreased in MRL/lpr mice compared with C57BL/6 J mice (Fig. 1b, d). All mice were 4 months old at the time of examination. Immunohistochemistry analysis was also used to validate renal B1R and B2R expression. Immunohistochemistry did not reveal renal B1R expression in C57BL/6 J mice (Fig. 2a), whereas B1R-positive staining was detected in the glomeruli and renal tubules of MRL/lpr mice (Fig. 2b). In contrast, B2R-positive staining was detected in glomeruli in C57BL/6 J mice (Fig. 2c); renal expression of B2R was decreased in MRL/lpr mice compared with the controls (Fig. 2d), consistent with the western blot results.

### The effect of bradykinin receptor B1R blockade on body weight and spleen and kidney weight in MRL/lpr mice

**Fig. 3:**
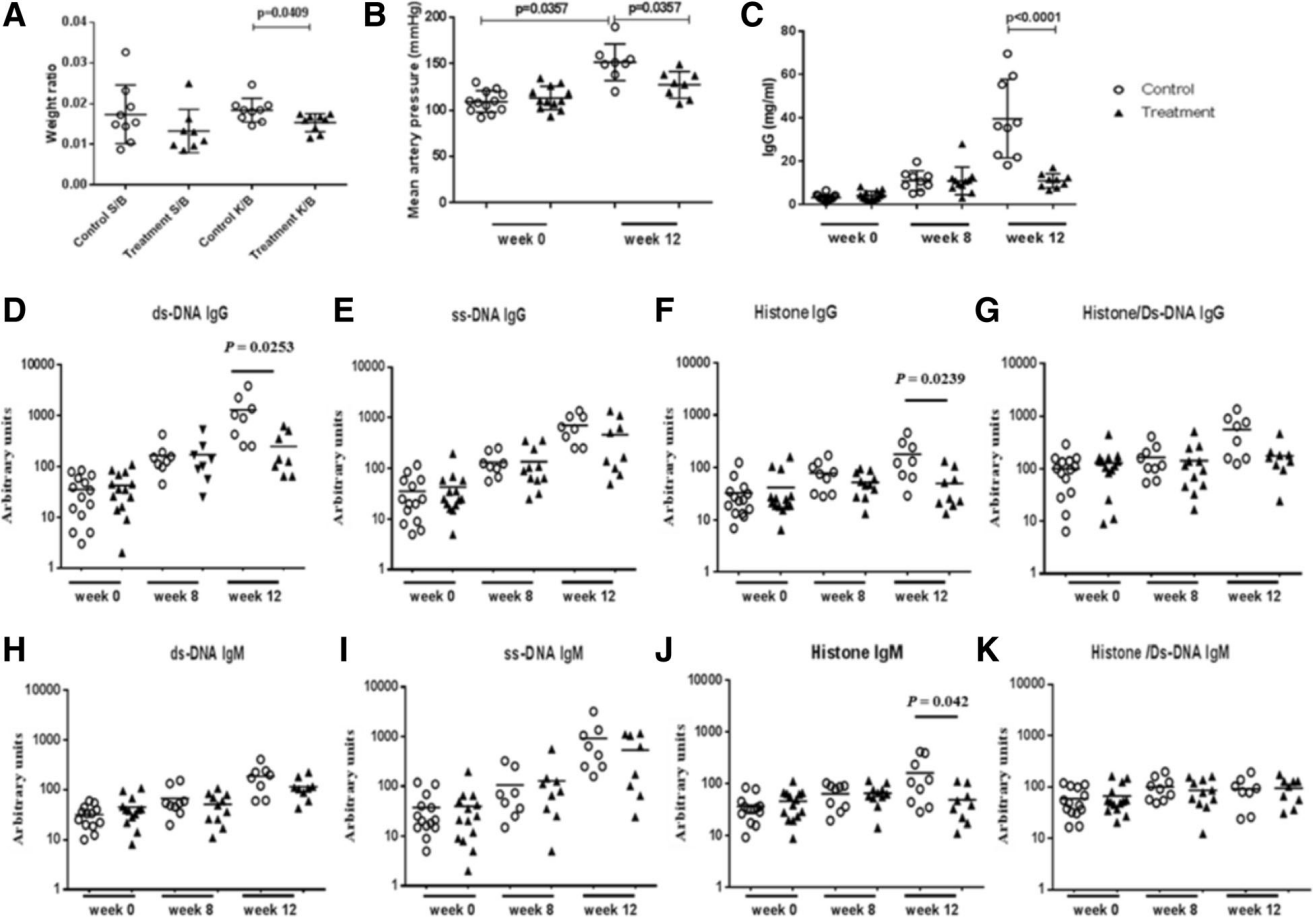
The effect of bradykinin 1 receptor (B1R) blockade on body weight, blood pressure, total serum IgG, and autoantibody levels. Twenty-eight 4-month-old MRL/lpr mice were divided into a control group (*n* = 14) and a treatment group (*n* = 14). The B1R antagonist SSR240612 was dissolved in water containing dimethyl sulfoxide (DMSO) to make a final concentration of 1.5 mg/mL in 0.9% DMSO. Mice in the treatment group were administered 10 mg/kg per day SSR240612 by gavage every other day, whereas the mice in the control group received 10 mg/kg per day 0.9% DMSO by gavage every other day. No significant difference was seen in the ratio of spleen weight to body weight between the control group and the treatment group (**a**). In addition, B1R blockade was associated with a decreased ratio of kidney weight to body weight compared with the control group (**a**, *P* = 0.0409). MRL/lpr mice showed higher blood pressure levels at 12 weeks than baseline. B1R blockade lowered the mean artery blood pressure compared with the control group (*P* = 0.0357, **b**). B1R blockade lowered total serum IgG levels after 12 weeks of treatment (**c**, *P* = <0.0001). Serum IgG anti-dsDNA, IgG anti-histone, and IgM anti-histone decreased significantly in the treatment group compared with the control group (**d**, *P* = 0.025; **g**, *P* = 0.024, **k**, *P* = 0.042). B1R blockade didn't affect IgG anti-ssDNA or IgM anti-ssDNA (**e**, **i**). A non-parametric Mann–Whitney test was used to analyze the data. Taking into account the missing values (for deceased animals), the corrected *P* values were as follows: **a**: *P* <0.006 for spleen/body ratio and *P* <0.0002 for kidney/body ratio, **b** (mean BP): *P* <0.0001, **c** (IgG): *P* <0.001; **d** (anti-DNA): *P* <0.0001; **f** (anti-histone): *P* <0.0001), **g** (anti-histone/DNA): *P* <0.0015; **h** (IgM anti-dsDNA): *P* <0.0026; **j** (IgM anti-histone): *P* <0.0002, and the remaining significance levels were unaltered

B1R blockade did not affect the body weight of MRL/lpr mice (Additional file 1: Figure S1). No significant difference was seen in the ratio of spleen weight to body weight between the control group and the treatment group (Fig. 3a). In addition, it was found that B1R blockade decreased the ratio of kidney weight to body weight compared with the control group (Fig. 3a, *P* = 0.0409).

### Bradykinin receptor B1R blockade reduced blood pressure in MRL/lpr mice

Kinins are involved in BP regulation. To ascertain whether B1R blockade can impact BP in MRL/lpr mice, we monitored the BP before and after B1R antagonist treatment (Fig. 3b). Baseline MAP BP levels were similar in the two groups of mice (104.3 ± 14.29 mm Hg versus 109.0 ± 21.85 mm Hg). The MAP of the control group of MRL/lpr mice was 155.8 ± 20.59 mm Hg at 12 weeks. B1R blockade lowered the MAP in treated MRL/lpr mice to 122.5 ± 10.00 mm Hg, which is significantly lower compared with the control group.

### Bradykinin receptor B1R blockade reduced circulating levels of autoantibodies and IgG

To assess the effect of B1R blockade on systemic immune response in MRL/lpr mice, we assayed total serum IgG levels and autoantibody levels after the B1R blockade. We found that B1R blockade lowered total serum IgG concentrations after 12 weeks of treatment (Fig. 3c, *P* <0.0001) but not total IgM (data not shown). IgG anti-dsDNA, IgG anti-Histone, and IgM anti-Histone antibodies were also decreased significantly in the treated mice compared with the control group (Fig. 3d, *P* = 0.025; Fig. 3g, *P* = 0.024, Fig. 3k, *P* = 0.042). However, the reduction in most of the other autoantibodies assayed attained statistical significance once the data were corrected for the missing values for the deceased animals at the 12-week time point (Fig. 3).

### Bradykinin receptor B1R blockade reduced proteinuria and serum BUN in MRL/lpr mice

**Fig. 4:**
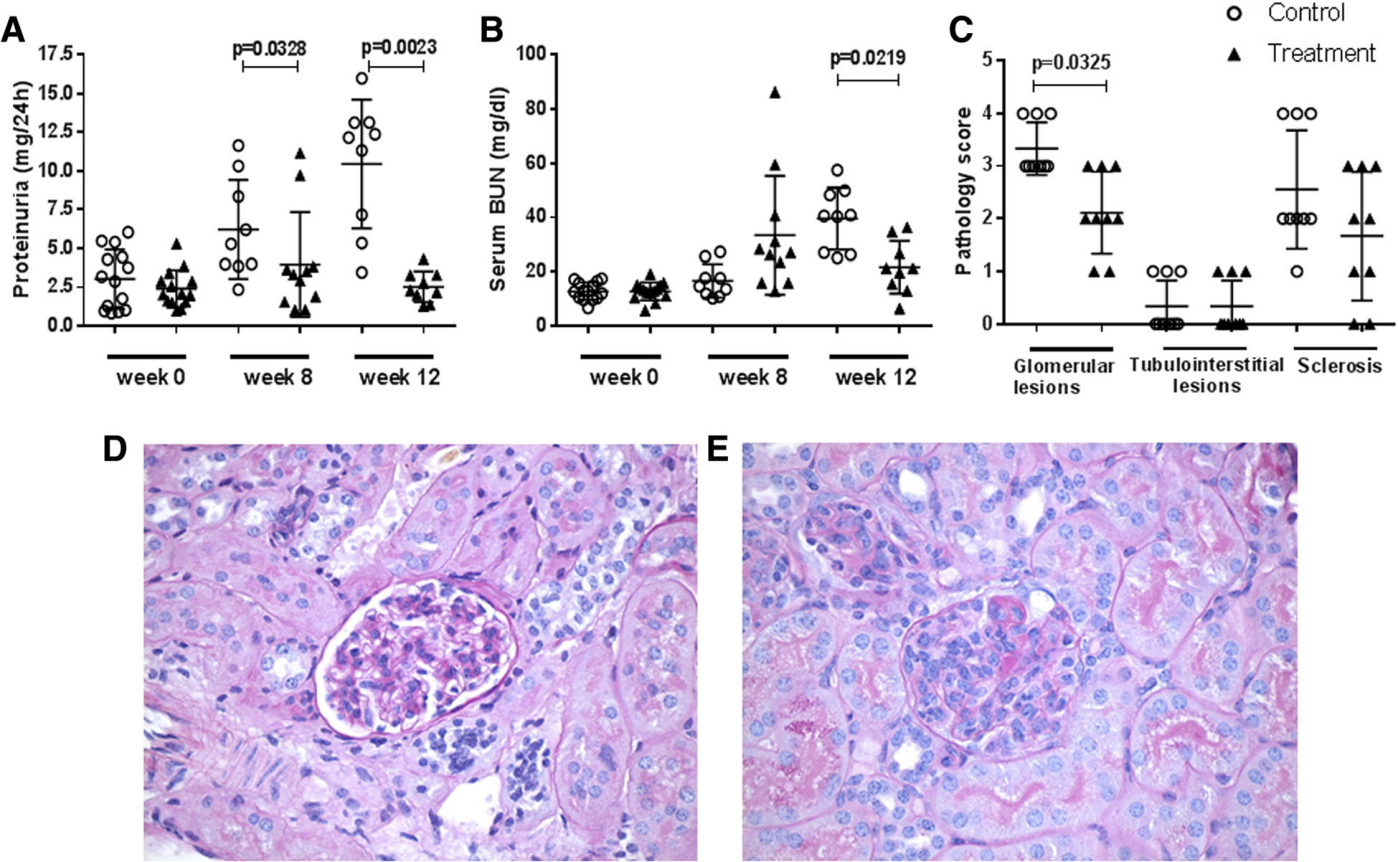
Proteinuria, renal function, and renal pathology lesions in bradykinin 1 receptor (B1R) blockade group and control group of MRL/lpr mice. Twenty-eight 4-month-old MRL/lpr mice were divided into a control group (*n* = 14) and a treatment group (*n* = 14) and administered the B1R antagonist SSR240612B1R or placebo for 12 weeks, as described above. B1R blockade ameliorated proteinuria (*P* = 0.0023, **a**) and serum BUN (*P* = 0.0219, **b**) levels in the treatment group compared with the control group. B1R blockade attenuated glomerular injury significantly (**c**, *P* = 0.0325). Glomerular sclerosis was reduced after B1R blockade, but there was no statistical difference between the two groups. A similar degree of interstitial lesions was seen between the two groups. Shown in (**d**) and (**e**) are representative images from periodic acid–Schiff–stained, formalin-fixed, paraffin-embedded renal sections from the control (**e**) and B1R inhibitor-treated (**d**) mice. (Original magnification 600×). The control group showed significant endocapillary cellular proliferation, membrane thickness, and irregularity in glomeruli, with obliteration of capillary lumina. A non-parametric Mann–Whitney test was used to determine statistical significance. Taking into account the missing values (for deceased animals), the corrected *P* values were as follows: **a**: *P* <0.0001, **b**: *P* <0.0001, **c**: *P* <0.0001 for glomerular lesions, *P* <0.014 for sclerosis, and not significant for TI score

In view of the increased renal expression of B1R in murine LN, we next investigated the effect of B1R blockade on proteinuria and renal function. B1R blockade diminished proteinuria 8 weeks after treatment (Fig. 4a, *P* = 0.0328). Both proteinuria and serum BUN were lower after 12 weeks of treatment compared with the control group (Fig. 4a, b, *P* = 0.0023, *P* = 0.0219), indicating that B1R blockade subdues LN.

### Bradykinin receptor B1R blockade regulated systemic and local intra-renal immune cell populations

To investigate possible mechanisms through which B1R blockade may be mitigating LN, splenocytes and intra-renal cells were isolated from the control group and the treatment group, and flow cytometry was used to interrogate the immune cell populations (Table 1). Total splenocytes were decreased after 12 weeks of B1R antagonist treatment. Absolute cell numbers of CD3^+^CD4^+^, B220^+^, and B220^+^CD21^high^CD23^low^ marginal zone B cells in spleens were also decreased in the treated mice compared with the control group. No significant difference was seen in total intra-renal immune cells between the two groups. It was also observed that the absolute cell numbers of intra-renal CD45^+^CD3^+^CD4^+^, CD45^+^CD11b^+^F4/80^+^, and CD45^+^ CD11b^+^Gr-1^+^ infiltrating cells were lowered after 12 weeks of B1R blockade compared with the control MRL/lpr mice (Table 1).

**Table 1:** Immune cells subsets in MRL/lpr mice

	MRL/lpr control mice	MRL/lpr + SSR treatment
Spleen: absolute cell numbers (×10^6^)		
Total cells	174.1 ± 16.8	88.90 ± 17.06**
Total T cells (CD3^+^)	63.15 ± 9.14	40.75 ± 19.97
CD3^+^ CD4^+^ cells	9.51 ± 1.13	5.04 ± 0.81**
CD3^+^ CD8^+^ cells	2.67 ± 0.75	1.56 ± 0.61
CD3^+^ CD4^+^ CD69^+^ cells	2.56 ± 1.28	1.76 ± 0.21
CD3^+^ CD4^+^ foxp3 cells	0.97 ± 0.21	0.59 ± 0.32
Total B cells (B220^+^)	104.5 ± 25.82	47.85 ± 16.62*
B220^+^ CD21^high^CD23^low^ cells	11.82 ± 1.86	5.94 ± 1.29**
B220^+^ CD23^high^CD21^low^ cells	6.48 ± 1.85	10.34 ± 6.31
CD11b^+^ F4/80^+^ CD86^+^ cells	1.80 ± 0.23	1.10 ± 0.74
CD11c^+^ CD86^+^ cells	9.72 ± 1.65	6.54 ± 2.50
Kidney: absolute cell numbers (×10^5^)		
Total cells	27.16 ± 5.73	18.97 ± 1.22
Total infiltrating cells (CD45^+^)	6.92 ± 1.63	4.75 ± 1.61
CD45^+^ CD3^+^ CD4^+^ cells	1.57 ± 0.29	0.91 ± 0.18*
CD45^+^ CD3^+^ CD4^+^ foxp3 cells	0.18 ± 0.05	0.17 ± 0.04
CD45^+^ CD11b^+^ F4/80^+^ cells	3.06 ± 1.27	1.04 ± 0.12*
CD45^+^ CD11c^+^ cells	3.52 ± 2.07	1.96 ± 0.94
CD45^+^ CD11b^+^ Gr-1^+^ cells	0.43 ± 0.07	0.20 ± 0.006*

*P <0.05; **P <0.01

### Bradykinin receptor B1R blockade prevented progression of renal disease

We next investigated the therapeutic effect of B1R blockade on renal lesions. Untreated MRL/lpr mice showed progressive renal injury (Fig. 4e). In contrast, treatment with the B1R antagonist ameliorated glomerular injury significantly (Fig. 4c, d, *P* = 0.0325). No difference in interstitial lesions was observed between the two groups of mice. Once the data were corrected for the missing data due to the deceased animals, the reduction in glomerulosclerosis scores also attained statistical significance (Fig. 4 legend). We next examined whether the reduced disease in the treated mice had an impact on survival. The survival times after treatment were 68 ± 21 days in the control group and 76 ± 14 days in treatment group (log-rank test *P* = 0.087). Thus, B1R blockade did not significantly increase the survival time of the lupus mice (Additional file 1: Figure S3).

### Bradykinin receptor B1R blockade reduced urine and renal chemokine expression

**Fig. 5:**
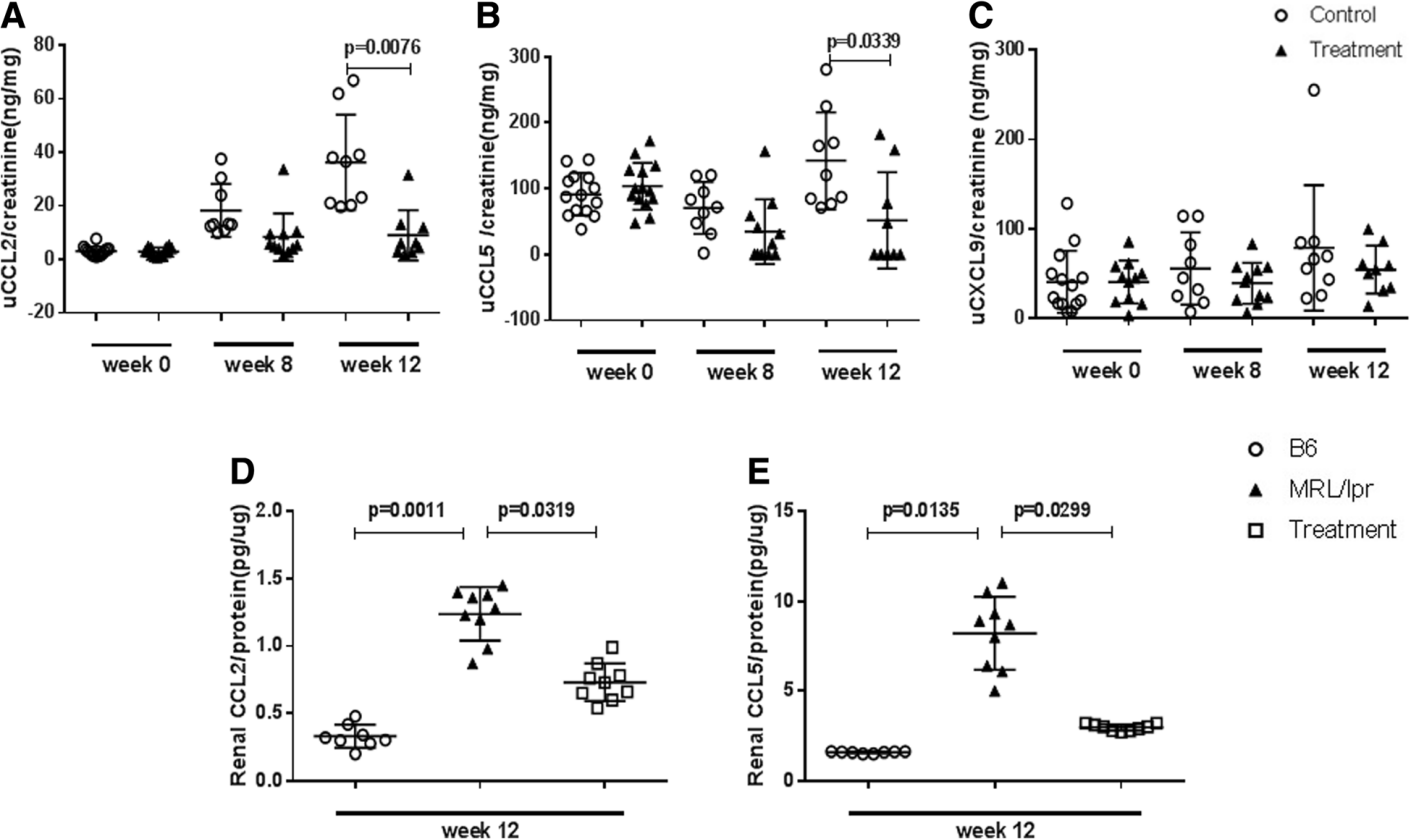
Urine and renal chemokine levels in bradykinin 1 receptor (B1R) blockade treatment group and control group of MRL/lpr mice. Twenty-eight 4-month-old MRL/lpr mice were divided into a control group (n = 14) and a treatment group (n = 14) and administered the B1R antagonist SSR240612B1R or placebo for 12 weeks, as described above. Urine CCL2, CCL5, and CXCL9 levels were assayed by ELISA. Urine CCL2 and CCL5 were elevated in MRL/lpr group and B1R blockade treatment decreased urine CCL2 and CCL5 levels after 12 weeks of treatment (**a**, *P* = 0.0076; **b**, *P* = 0.0339). Urine CXCL9 levels showed no significant difference between the two groups (**c**). Renal CCL2 and CCL5 levels were elevated in MRL/lpr mice compared with B6 mice at 12 weeks (**d**, *P* = 0.0011; **e**, *P* = 0.0135). The B1R blockade treatment group revealed lower renal CCL2 and CCL5 levels than the control group (**d**, *P* = 0.0319; **e**, *P* = 0.0299). A non-parametric Mann–Whitney test was used to determine statistical significance. Taking into account the missing values (for deceased animals), the corrected *P* values were as follows: **a**: *P* <0.0001, **b**: *P* <0.0003, **c**: not significant; **d**: *P* <0.0001; and **e**: *P* <0.0001

To ascertain the effect of B1R blockade on renal chemokine expression, we used ELISA to assay levels of urine CCL2, CCL5, and CXCL9, chemokines reported to be elevated in MRL/lpr lupus mice. Compared with the healthy counterparts, urine CCL2 and CCL5 were increased in MRL/lpr mice; however, B1R blockade decreased urine CCL2 and CCL5 levels after 12 weeks of treatment (Fig. 5a, *P* = 0.0076; Fig. 5b, *P* = 0.0339). B1R blockade did not affect urine CXCL9 levels (Fig. 5c). To explore intra-renal chemokine expression, we measured renal lysate CCL2 and CCL5 levels by ELISA. Renal CCL2 and CCL5 levels were elevated in MRL/lpr mice compared with B6 mice (Fig. 5d, *P* = 0.0011; Fig. 5e, *P* = 0.0135). B1R blockade significantly decreased renal CCL2 and CCL5 levels compared with those of the control MRL/lpr mice (Fig. 5d, *P* = 0.0319; Fig. 5e, *P* = 0.0299) and these reductions became even more significant once the data were corrected for the missing values due to the deceased mice (Fig. 5 legend).

### Screening for potential side effects of therapy

As described earlier, B1R blockade did not significantly impact the body weight of the treated mice (Additional file 1: Figure S1). To monitor the effect of B1R blockade on liver function, serum ALT and AST were measured. The ALT and AST levels were similar between the control group and treatment group at 0 and 12 weeks (Additional file 1: Figure S2A and S2B).

## Discussion

Kallikreins catalyze the production of kinins from kininogens. Kallikreins and kinins exert multiple biological functions, including the regulation of cytokine release, pain, edema, leukocyte recruitment, and cell proliferation [13]. In our previous studies, we have reported that kallikrein gene polymorphisms are associated with lupus and nephrotoxic serum-induced nephritis [14, 15]. In addition, kallikreins play a reno-protective role in LN and nephrotoxic serum-induced nephritis [16]. However, the molecular mechanisms for this reno-protection have not been systematically investigated.

It is known that kallikreins promote the generation of bradykinins, BK and BK-des-Arg9. Whereas BK binds to the B2R bradykinin receptor, BK-des-Arg9 binds to and activates another bradykinin receptor, B1R, particularly under inflammatory conditions [17]. B1R is expressed at inflammatory sites, induced by inflammatory mediators, such as interleukin-1 (IL-1) and tumor necrosis factor-alpha (TNF-α), in a nuclear factor-kappa B (NF-κB)- and mitogen-activated protein kinase (MAPK)-dependent manner [18, 19]. In particular, the B1R appears to play a key role in inflammation, pain, and fibrosis and has been implicated in inflammatory bowel disease, multiple sclerosis, and experimentally induced nephritis [1, 5, 20, 21]. Moreover, the B1R is expressed along the nephron and is involved in renal inflammation and fibrosis in other renal disease models [5, 22].

Along the same line, Pereira et al. [3] reported that B1R agonist exacerbated experimental focal and segmental glomerulonephritis (FSGS) but that B1R antagonist reduced proteinuria and glomerulofibrosis, reversed podocyte dysfunction, and played a protective role in the pathogenesis of FSGS. It has been documented that B1R staining was positive in renal tissue of patients with antineutrophil cytoplasmic antibody (ANCA)-associated vasculitis and Henoch–Schönlein purpura nephritis [5]. B1R blockade or ablation was also documented to be effective in ameliorating renal fibrosis in experimental obstructive nephropathy [7], reducing the renal inflammatory response in cisplatin or LPS-induced acute renal injury and ischemic-reperfusion injury in murine models [6, 23, 24]. In our previous work in anti-GBM–induced experimental nephritis mice, blockade of the B2R bradykinin receptor worsened disease whereas blockade of B1R ameliorated disease [15], once again suggesting the pro-inflammatory and pathogenic role of the B1R in immune-mediated nephritis. Considering those previous studies, we also speculated that B2R may mediate a reno-protective effect once engaged by BK in autoimmune nephritis. The goal of this study is to extend these findings to LN.

Our study demonstrated that renal B1R expression was increased but that B2R expression was decreased in MRL/lpr lupus-prone mice. More importantly, renal B1R:B2R ratios were significantly increased and this could significantly skew the effects of kinins to be mediated primarily via pro-inflammatory pathways. Interestingly, no literature has been reported on end-organ B1R:B2R ratios in any disease model. Given the accumulating evidence that B1R may be pro-inflammatory and B2R may be reno-protective, renal B1R:B2 ratios may be an important determinant of renal inflammation, possibly with diagnostic or disease-predictive potential. Our study also demonstrates that disease amelioration in MRL/lpr mice may be associated with reduced intra-renal chemokine expression and immune infiltrates.

A number of chemokines have been shown to play important roles in LN. Renal CCL2, CCL5, and CXCL9 mRNA and protein have been reported to be elevated in MRL/lpr mice and the roles of these chemokines and their corresponding receptors have been documented in murine LN [25–27]. It has been speculated that B1R may contribute to disease by regulating inflammatory cytokines, such as CCL2, MIP-1, and CCL5, in experimental FSGS [3]. SSR240612 was initially reported as a novel non-peptide antagonist of the B1R with selectivity for B1R versus B2R in the range of 500- to 1000-fold, where SSR240612 inhibited BK-des-Arg9 induced inositol monophosphate formation in human fibroblast MRC5 with a half maximal inhibitory concentration (IC_50_) of 1.9 nM [28]. Subsequently, SSR240612 has been successfully used as a B1R antagonist in animal models of other renal and non-renal diseases [5, 29, 30]. B1R blockade has also been reported to reduce renal inflammation by downregulating renal CCL2, CCL5, and CCL7 in the anti-GBM nephritis model [5]. In addition, renal CCL2 and CCL7 overexpression was observed in an obstructive nephropathy model and B1R antagonist was demonstrated to inhibit renal inflammation and fibrosis *in vivo* and *in vitro*, partially mediated by inhibiting CCL2 and CCL7 expression [25]. Consistent with the earlier reports, B1R blockade in murine LN is also associated with downregulation of renal chemokines, notably CCL2 and CCL5, in the present study. Consistent with the chemokine reduction within the kidneys, B1R blockade in MRL/lpr mice also reduced intra-renal levels of CD45^+^ CD3^+^CD4^+^ T cells, CD45^+^ CD11b^+^F4/80^+^ macrophages, and CD45^+^ CD11b^+^Gr-1^+^ granulocytes, possibly driven in part by alterations in CCL2 and CCL5 expression [31].

To our surprise, B1R blockade not only subdued LN but also suppressed systemic autoimmunity, as noted by the reduction in total serum IgG and anti-dsDNA IgG levels, as well as splenic marginal zone B cells and CD4^+^ T cells. It has been reported that B1R expression was upregulated on T cells from peripheral blood of patients with multiple sclerosis [20, 32]. Moreover, B1R expression has been reported on dendritic cells [33]. Although B1R expression on systemic immune cells in LN was not examined in this study, our results support the hypothesis that bradykinin receptor blockade may serve to ameliorate systemic immunity by silencing dendritic cells and helper T cells, which in itself should be sufficient to ameliorate lupus, both at the systemic level and the end-organ disease manifestations, including LN.

Our study also revealed that B1R blockade reduced BP in MRL/lpr mice. Previously, it has been reported that B1R blockade exerts a BP-lowering effect in hypertensive rat models [29, 34]. It has also been reported that brain B1R antagonist decreased BP via a raclopride sensitive mechanism by downregulation of dopaminergic pathways which otherwise may have hypertensive effects [29]. Additionally, it has been reported that activation of B1R elevates superoxide anions by activating NADPH oxidase in the vasculature; moreover, B1R antagonist treatment was shown to decrease high BP in 12-week glucose-fed rats by reducing oxidative stress [30]. It has been shown that excess of either superoxide or hydrogen peroxide in the renal medulla reduces renal medullary blood flow and enhances Na^+^ reabsorption and hypertension [35]. B1R blockade in the present study may have reduced BP in MRL/lpr mice via several different mechanisms. Indeed, other mechanisms not related to the bradykinin pathway may also have indirectly contributed to the reduction in BP but this warrants further investigation. Although hypertension is not a feature of lupus autoimmunity, a substantial fraction of patients with SLE and LN also have hypertension as a comorbidity. Thus, the anti-hypertensive effect of B1R blockade constitutes an additional beneficial impact of this therapeutic modality in LN.

Despite the improvement in autoimmunity, LN, and hypertension, there was no significant difference in survival following B1R blockade. However, the observation that treated mice survived 8 days longer on average (*P* <0.087) warrants a repeat of these studies with a larger animal cohort or longer treatment duration or both. In addition, the potential impact of increased drug dosage needs to be examined.

## Conclusions

In summary, these studies have yielded several novel observations. First, there is a dramatic imbalance in bradykinin receptor expression within LN kidneys, and significantly heightened B1R:B2R receptor ratios resulted in increased levels of the pro-inflammatory B1R. Second, these studies show that targeting the bradykinin B1Rs may offer therapeutic benefit in three different ways: (a) amelioration of systemic lupus and dampening of systemic autoimmunity and autoantibodies, (b) reduced LN, possibly mediated by lowering of chemokines and intra-renal immune infiltrates, and (c) reducing the BP. Further studies are clearly warranted to explore the utility of bradykinin B1R blockade in patients with SLE given the multiple avenues through which this axis impacts disease.

## Additional file

Additional file 1: **Figure S1.** Body weight in the control group and treatment group of MRL/lpr mice. **Figure S2.** Liver function in the control group and treatment group of MRL/lpr mice. **Figure S3.** Impact of bradykinin 1 receptor (B1R) blockade on mortality in MRL/lpr mice. (DOCX 83 kb)

## Abbreviations

ALT: Alanine aminotransferase
AST: Aspartate aminotransferase
B1R: Bradykinin 1 receptor
BP: Blood pressure
BUN: Blood urea nitrogen
DMSO: Dimethyl sulfoxide
ELISA: Enzyme-linked immunosorbent assay
FSGS: Focal and segmental glomerulonephritis
GBM: Glomerular basement membrane
HRP: Horseradish peroxidase
LN: Lupus nephritis
LPS: Lipopolysaccharide
MAP: Mean artery pressure
SLE: Systemic lupus erythematosus
