# Supplementary material for: Bradykinin 1 receptor blockade subdues systemic autoimmunity, renal inflammation, and blood pressure in murine lupus nephritis
Source: Arthritis Res Ther. 2019 Jan 8;21:12. doi: 10.1186/s13075-018-1774-x (PMC6325757; doi:10.1186/s13075-018-1774-x)

**Figure S1**. **Body weight in the control group and treatment group of MRL/lpr mice**. B1R blockade did not significantly affect body weights in the treated mice.

**Figure S2**. **B1R blockade did not impact liver function.** To monitor the effect of B1R blockade on liver function, serum ALT and AST were measured. The ALT (2A) and AST (2B) levels were similar between the control group and treatment group of MRL/lpr mice at 0 week and 12 weeks.

**Figure S3.** **Impact of** **B1R blockade on mortality in MRL/lpr mice.** The average survival time (after treatment) was 68 days in the control group and 76 days in the B1R blockade treatment group of mice (Kaplan Meier survival analysis, log rank test *P* =0.087). Thus, B1R blockade did not reduce mortality in MRL/lpr mice.

Qin et al., Fig. S1

Qin et al., Fig.2

**B**

**A**

Qin et al., Fig. S3


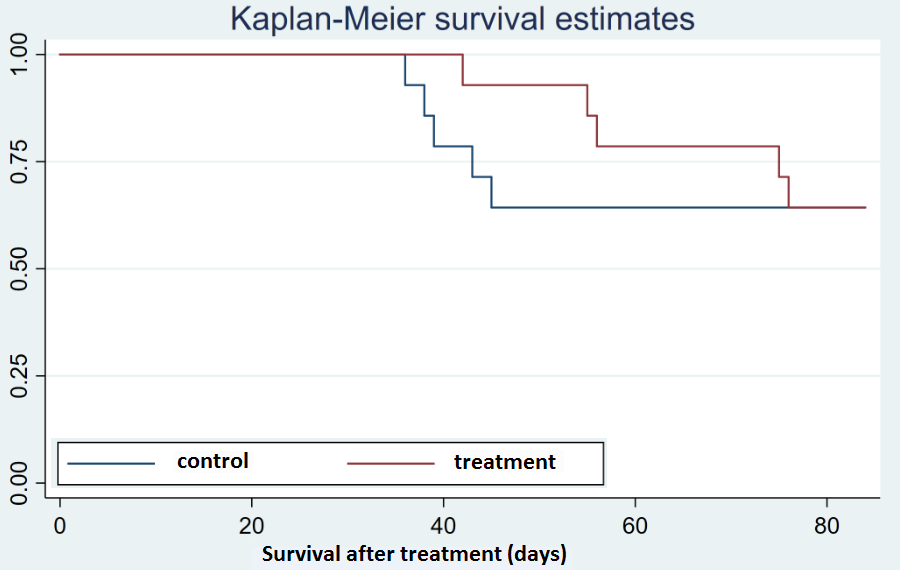

Supplement: Supplementary file 1 — Figure S1. Body weight in the control group and treatment group of MRL/lpr mice. Figure S2. Liver function in the control group and treatment group of MRL/lpr mice. Figure S3. Impact of bradykinin 1 receptor (B1R) blockade on mortality in MRL/lpr mice. (DOCX 83 kb) [file 13075_2018_1774_MOESM1_ESM.docx]
